# An Interpretable Deep Learning System for Fine-Grained Classification and Longitudinal Tracking of Neonatal Auricular Deformities

**DOI:** 10.3390/biology15130985

**Published:** 2026-06-23

**Authors:** Yihui Feng, Xujun Hu, Xiwen Zhang, Xiaobao Ma, Jialin Xie, Jianyong Chen, Yangyang Yuan

**Affiliations:** 1 School of Medical Technology and Information Engineering, Zhejiang Chinese Medical University, Hangzhou 310053, China; fengyihui1106@163.com (Y.F.);; 2 Department of Otorhinolaryngology-Head and Neck Surgery, Xinhua Hospital, Shanghai Jiaotong University School of Medicine, Shanghai 200092, China; 3 The Second School of Clinical Medicine, Zhejiang Chinese Medical University, Hangzhou 310053, China

**Keywords:** neonatal auricular deformities, deep learning, disease severity scoring, supervised contrastive learning, fine-grained classification, interpretable AI

## Abstract

Early non-invasive correction of neonatal auricular deformities is highly dependent on timely and precise diagnosis. However, clinical practice is often compromised by the subjectivity of visual assessments and the lack of objective tracking metrics, which frequently leads to missed optimal treatment windows. To address these challenges, we developed an interpretable deep learning-based diagnostic system for the automated screening and fine-grained classification of these deformities. Methodologically, a large-scale, multi-source dataset (*n* = 4644) was curated to support model training. The system pairs an automated object detector (YOLOv11) for background-reduced region-of-interest isolation with a cascaded classification pipeline optimized via ConvNeXt-Tiny. Crucially, we introduced a supervised contrastive learning module to project high-dimensional morphological features into a continuous severity score, enabling quantitative longitudinal tracking of therapeutic efficacy. To evaluate generalization and robustness, the framework underwent rigorous evaluation across three independent real-world cohorts and one controlled synthetic stress test. The system achieved 88.2% accuracy (Area Under the Curve (AUC): 0.949) in binary screening and 87.4% accuracy (macro-AUC: 0.976) in multi-class subtyping on the internal baseline. To enhance interpretability and build clinical trust, Gradient-weighted Class Activation Mapping (Grad-CAM) was utilized to explore the spatial distribution of the model’s attention, which frequently aligned with key anatomical landmarks. Furthermore, the learned severity scores robustly quantified post-intervention improvements (*p* = 0.0004), effectively capturing subtle anatomical normalization. While validation for rare subtypes remains underpowered, and the severity score currently functions mainly as a learned morphological similarity index requiring future clinical calibration, this study ultimately provides an objective and standardized web-based tool to facilitate the early intervention and precision management of neonatal auricular anomalies.

## Simple Summary

At present, the clinical assessment of the auricle morphology of neonates only relies on subjective decisions, which often leads to neonates missing the short time window for early non-surgical treatment. Furthermore, there is currently no objective method to track the effectiveness of its treatment. To solve these problems, we developed a deep learning based decision-making and evaluation system to automatically diagnose and track ear conditions from basic photographs. The results show that our system can accurately screen for abnormal ears and identify six specific types of ear malformations. In addition, the deformity degree score can effectively assess the improvement before and after treatment. In conclusion, an easy-to-use web tool based on this system was developed to assist clinicians in making rapid diagnoses and tracking treatment outcomes, thereby improving clinical decision-making and patient outcomes.

## 1. Introduction

Auricular deformities are common in newborns, with reported incidence rates as high as 57.46%. Although a significant proportion of these anomalies may resolve spontaneously [1], the unpredictability of self-correction underscores the necessity of early identification and timely intervention for persistent cases [2,3,4]. Despite clinical advances, substantial challenges continue to impede the early and accurate diagnosis in routine practice. First, high rates of missed diagnosis and misdiagnosis are driven by insufficient parental awareness [5], strong diagnostic subjectivity among doctors [6], and a lack of experience in primary medical institutions [7]. Consequently, most newborn patients miss the optimal 2–3-month intervention period for non-invasive correction [8], failing to leverage the high plasticity of auricular cartilage during this optimal therapeutic window [9,10]. Second, morphological subtypes are highly complex; for instance, certain malformations like lop ear and Stahl’s ear require professional judgment and intervention within 1–3 weeks after birth [11] to achieve corrective success rates up to 93.9% [9]. Third, while advanced imaging technologies improve detection rates [12], their high cost and substantial time requirements preclude widespread routine clinical screening. Therefore, the development of an accurate, efficient, and readily accessible diagnostic method for neonatal auricular deformities is particularly necessary to enhance diagnostic performance, halt disease progression and improve long-term prognosis.

With the rapid development of medical artificial intelligence, deep learning has brought significant advances to medical image analysis, frequently matching or exceeding diagnostic standards set by human experts [13,14]. Several deep learning models have been applied to auricular deformity analysis, including critical tasks such as binary screening [15], reconstruction outcome evaluation [16], and severity grading for specific subtypes like microtia [17]. Most recently, Ren et al. developed a smartphone-based application integrating a pre-trained ResNet50 model to perform both binary screening and multi-class classification [18]. While these previous models collectively illustrate the promising applicability of deep learning, significant translational challenges persist at both the data and model levels [15,16,17,19]. First, a critical bottleneck lies in the data scale and quality; most existing studies predominantly rely on small-scale, single-center datasets or imbalanced public resources such as BabyEar4K [20], where limited sample sizes and severe inter-class imbalances inherently lead to poor generalization and classification bias [18]. Second, these data constraints, combined with the structural complexity from binary to multi-class classification, result in suboptimal model performance. Third, the inherent “black-box” nature of deep learning compromises predictive interpretability, reducing clinical credibility and restricting widespread promotion and application [13].

In this study, we propose a severity scoring-centered intelligent diagnostic system for neonatal auricular deformities. We hypothesized that integrating a multi-source dataset with a cascaded classification pipeline and a contrastive learning-based severity scoring system would improve fine-grained subtype recognition and enable quantitative longitudinal tracking of treatment outcomes, addressing a continuous quantification dimension currently missing in clinical practice. A key contribution of this study is the development of a continuous, contrastive learning-based severity index, addressing the critical clinical void of objective longitudinal tracking. To translate this core algorithm into a viable clinical workflow, we engineered a cascaded pipeline acting as essential supporting modules. Specifically, a YOLOv11 detector was employed to eliminate complex background interference common in primary care photos, while a ConvNeXt-Tiny backbone ensured robust feature extraction for morphologically ambiguous boundaries. Furthermore, a conditional diffusion model (DDPM) was utilized solely as a stress test mechanism to simulate rare clinical scenarios [21]. Finally, to facilitate seamless clinical integration, the entire pipeline—from automated cropping to fine-grained subtyping and severity quantification—was embedded into a user-friendly web workstation, providing clinicians with one-click, highly interpretable diagnostic evidence via Grad-CAM heatmaps [22].

## 2. Materials and Methods

### 2.1. Image Dataset Collection

A large-scale, multi-source image repository was curated from three real world sources (a public benchmark dataset, a literature-derived collection and a prospective clinical dataset) alongside a controlled synthetic set. The dataset comprises two primary categories (normal and abnormal auricles) with the latter further stratified into six clinical subtypes: lop ear, Stahl’s ear, helical deformity, cup/constricted ear, cryptotia, and microtia. Clinically, this taxonomy strictly encompasses both auricular deformities (characterized by the abnormal folding of fully developed cartilage) and auricular malformations (characterized by tissue hypoplasia, notably microtia). In this study, cup and constricted ears were grouped together as they represent a continuous morphological spectrum of the same upper pole deficiency, and this unified grouping aligns with their shared non-invasive treatment pathways and prevents boundary confusion during deep feature extraction. Specifically, the foundational cohort was derived from the BabyEar4K open-source repository, contributing 3852 bilateral frontal photographs from 1926 neonates. To enrich the diversity of malformation representations, we conducted a systematic literature search, integrating 688 high-quality images from published case reports (https://doi.org/10.5281/zenodo.20672605) after rigorously excluding those with low resolution, duplicated publications, or inconsistent photographic angles. Dataset labels were derived from article identifiers and verified by experts to ensure diagnostic accuracy and taxonomic consistency. Furthermore, all literature-derived images were integrated in strict compliance with academic fair use guidelines for non-commercial research and open-access policies (e.g., CC-BY/CC-BY-NC), with proper attribution maintained. All images underwent manual cropping to extract the auricles while strictly eliminating facial features. Notably, this dataset was reserved solely for model training purposes. Furthermore, to conduct a feasibility experiment assessing model robustness on rare subtypes, a generative library was synthesized using the conditional diffusion model to serve as a simulated stress test (Section 2.2). Finally, a prospective clinical dataset was established, which encompassed 81 affected auricles from 61 neonates with standardized pre- and post-treatment frontal views. The detailed composition and demographic distribution across all sources are summarized in Table 1.

### 2.2. Simulating Rare Clinical Scenarios via Synthetic Data Generation

To evaluate the feasibility of the proposed framework under simulated stress conditions, we employed a class-conditional Denoising Diffusion Probabilistic Model (DDPM) to generate candidate synthetic images for each deformity subtype. To ensure the high fidelity and morphological realism of the generated data, a rigorous quality control pipeline was implemented. Quantitatively, the generation model achieved a Fréchet Inception Distance (FID) of 159.40 and an Inception Score (IS) of 3.06 ± 0.09. Qualitatively, the generated images were subjected to rigorous visual inspection to ensure that the auricles closely resembled true clinical proportions without severe surreal pixel artifacts. This review focused primarily on two core dimensions: first, the accurate representation of subtype-specific anatomical hallmarks; and second, the overall morphological plausibility. Representative failure cases excluded during this review process are provided in Figure S1.

Furthermore, to address the limited real examples of rare subtypes (e.g., cryptotia and microtia), a class conditional DDPM was implemented utilizing a shared latent space via cross attention, coupled with dynamic data augmentation. This allowed minority classes to leverage foundational morphological priors from abundant classes, effectively mitigating overfitting and expanding intra-class diversity. Detailed implementation parameters, including network architecture and training schedules, are provided in Table S1. Ultimately, a strictly balanced subset of 180 high-quality synthetic images (30 per subtype) were selected to form the controlled stress test set.

### 2.3. Training and Test Dataset Construction

To ensure rigorous model development and unbiased performance evaluation, we implemented distinct data partitioning strategies for the binary screening and six-class subtype classification tasks. For the binary screening task, a comprehensive pool of 4383 images was constructed by merging all normal images from BabyEar4K (*n* = 2136) and abnormal images from both BabyEar4K (*n* = 1559) and the literature-derived repository (*n* = 688). The combined dataset was split into training (80%, *n* = 3503) and internal test (20%, *n* = 880) sets using class-stratified random sampling (Table S2). For the six-class subtype classification task, a multi-stage validation framework was adopted (Table S2). First, the literature-derived dataset was randomly split into two subsets: 80% (*n* = 550) for model integration and 20% (*n* = 138) as a strictly held-out literature test set. The integration subset was then fused with 1559 abnormal images from BabyEar4K to form an aggregated dataset (*n* = 2109). This dataset was further divided into a training set (*n* = 1697) and an internal test set (*n* = 412) using stratified sampling to maintain consistent subtype proportions. Crucially, the Synthetic stress test set (Section 2.2) and the prospective Clinical test set (Section 2.1) served as two distinct evaluation cohorts for the six-class task. It should be noted that the synthetic set is not a true external validation; rather, it is a stress test designed to assess model robustness on a balanced set of rare subtypes. It should be noted that both internal test sets were utilized for architecture selection and subsequent pipeline tuning, the final metrics derived from it represent optimized model development performance rather than a strictly independent validation.

### 2.4. Data Preprocessing

To train the YOLOv11 detector, we utilized the high-quality bounding box annotations provided by the BabyEar4K dataset for all 3852 images. To ensure robust model optimization and evaluation, this dataset was partitioned into training (70%, *n* = 2696), validation (20%, *n* = 770), and testing (10%, *n* = 386) subsets. Notably, while all raw images are typically preprocessed via the YOLOv11 detector to isolate the auricular region and remove background interference, this module was bypassed during model training due to the highly curated, high-quality nature of our training set (see details in Table S3). For all other subsequent input images, however, YOLOv11 extraction is configured to be active by default. Subsequently, the cropped real images and the synthetic images were uniformly resized to 224 × 224 pixels to align with the input dimension requirements of the downstream feature extractors. Following spatial standardization to mitigate overfitting and enhance model generalization, a data augmentation pipeline was exclusively implemented during the training phase, including random horizontal flipping, random rotation and color jittering (brightness, contrast, and saturation factors). Finally, pixel intensity normalization was applied. Images in the validation and test sets were subjected only to cropping, resizing, and normalization.

### 2.5. Comparative Evaluation of Deep Learning Architectures

To investigate the suitability of different architectural paradigms for the fine-grained morphological classification of neonatal auricles, we systematically compared seven representative deep learning networks: ConvNeXt-Tiny, ResNet-50, EfficientNet-B3, MobileNetV3-Large, DenseNet-121, Swin Transformer-Tiny, and ViT-Base. All candidate networks were initialized with ImageNet-1K pre-trained weights. The original fully connected classification heads were substituted with task-specific linear layers, configured to output 2 dimensions for binary screening and 6 dimensions for deformity subtyping. Detailed hyperparameters (e.g., initial learning rate, batch size, and scheduler parameters) are provided in Table S4.

### 2.6. Model Evaluation and Comparison

A multi-dimensional suite of evaluation metrics was employed for model evaluation. For the six-class task, we calculated overall accuracy, macro-averaged precision, macro-averaged sensitivity, macro-averaged F1-score, macro-averaged Area Under the Receiver Operating Characteristic curve (ROC-AUC), and macro-averaged Area Under the Precision–Recall curve (PR-AUC). In addition, category-specific sensitivity, specificity, and precision were additionally reported to gauge the model’s discriminatory power for rare subtypes. For the binary screening task, performance was assessed via accuracy, sensitivity, specificity, precision, F1-score, ROC-AUC, and PR-AUC. To ensure statistical reliability, 95% confidence intervals (CIs) for all performance indicators were estimated using the non-parametric bootstrap method with 1000 resamples on the test sets. Systematic comparisons of the seven candidate models were conducted primarily based on the internal test set. Specifically, the macro-averaged F1-score and ROC-AUC were designated as the primary benchmarks for the six-class task to ensure equal weighting across all deformity categories, while accuracy and ROC-AUC served as the lead metrics for the binary screening task.

### 2.7. Visual Interpretability and Error Analysis via Grad-CAM

To enhance the transparency of the diagnostic model and elucidate the underlying anatomical drivers of misclassifications, Gradient-weighted Class Activation Mapping (Grad-CAM) was employed to generate visual attention heatmaps, localizing the most discriminative anatomical regions contributing to the model’s predictions. By comparing these focal regions between correctly and incorrectly classified instances, we qualitatively assessed the spatial association between the model’s attention and clinically relevant morphological variations versus background artifacts.

### 2.8. Deformity Severity Scoring System

To quantify the severity of auricular deformities, we developed a continuous scoring system based on supervised contrastive learning. A pre-trained ResNet-50 was utilized as the encoder, with its classification head replaced by a projection head mapping the 2048-dimensional features into a 128-dimensional contrastive space, followed by L2 normalization. This model was trained using a total of 4383 images, consisting of 2136 normal samples from BabyEar4K and 2247 abnormal samples covering six types of deformities (1559 of which came from BabyEar4K and 688 from the literature). Training details are provided in Table S5. Post training, a “Normal Auricle Prototype” was established by calculating the L2-normalized mean feature vector of all normal samples.

For any new input image, its L2-normalized feature vector was extracted to compute the cosine similarity with the normal prototype. This similarity metric was linearly scaled into an initial score s (ranging from 0 to 100), where a higher score indicates closer morphological proximity to the normal baseline:

*s* = (cosine_similarity + 1) × 50 (1)

To align this mathematical similarity with the non-linear clinical perception of deformity severity, the initial score s was converted into a final deformity severity score, D(*s*), using a calibrated piecewise function:

(2) $$D\left(s\right)=\left\{\begin{array}{c} \left(\frac{100-s}{25}\right)\times 20,\;if\;s\geq 75\\ 20+\left(\frac{75-s}{20}\right)\times 65,\;if\;55\leq s<75\\ 85+\left(\frac{55-s}{55}\right)\times 15,\;if\;s<55 \end{array}\right.$$

The piecewise thresholds (*s* = 55 and *s* = 75) were quantitatively anchored to the training baseline distributions. Specifically, milder subtypes (helical, Stahl’s, and lop ears) averaged 80.7, 77.5, and 76.6, respectively, whereas profound anomalies (cryptotia and microtia) averaged 65.0 and 61.7 (Table S6). Based on D(*s*), severity was categorized into five clinical tiers: Normal (≤15), Mild (16–35), Moderate (36–60), Severe (61–85), and Profound (>85). The Normal threshold (≤15) provides a conservative buffer for healthy controls (mean similarity: 85.6, translating to a D(*s*) of 11.5), effectively minimizing over-diagnosis of benign physiological variations. Conversely, the profound threshold (*s* > 85) is mathematically driven by the third segment (*s* < 55) of the piecewise function, strictly isolating severe anomalies that mandate urgent clinical intervention.

To assess the above deformity severity scoring system, we constructed a dedicated subset based on the prospective Clinical test set mentioned above (*n* = 81). After excluding samples with severe motion artifacts or incomplete records, we established a refined cohort containing 73 pairs of images matched before and after treatment to evaluate the sensitivity of this module to morphological changes. Table S2 provides detailed data distribution across all partitions.

### 2.9. Prototype Development of an Integrated Diagnostic Workstation

To translate the validated algorithms into an accessible clinical tool, we engineered a web-based diagnostic workstation. The system seamlessly integrates our complete end-to-end pipeline: automated ROI extraction via YOLOv11, fine-grained anomaly classification via ConvNeXt-Tiny, and quantitative severity scoring via the ResNet-50 prototype module. The platform provides an intuitive interface for clinicians to perform efficient batch uploads, receive real-time multimodal feedback (diagnostic categories, continuous severity scores, and Grad-CAM heatmaps). Currently operating as a research prototype, the system is deployed on a dedicated local GPU server. To ensure data security and comply with medical privacy standards, all data transmissions are encrypted. Uploaded images are processed strictly in memory and are immediately deleted post inference. The uploaded data will not be used for re-training.

## 3. Results

### 3.1. Assessment of Model Selection and Training Dynamics

**Figure 1:**
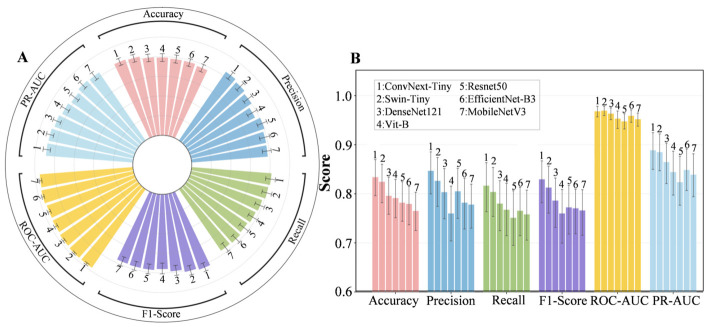
Performance comparison of different deep learning backbones in the intelligent diagnosis task of neonatal ear deformities. (**A**) A polar chart of performance evaluation for the binary abnormality screening task. This panel compares seven candidate deep learning models (ConvNeXt-Tiny, Swin Transformer Tiny, DenseNet-121, ViT-Base, ResNet-50, EfficientNet-B3, and MobileNetV3) across six metrics, with error bars representing 95% confidence intervals. (**B**) A bar chart of performance evaluation for the six-class deformity subtype identification task. It illustrates the comparison of the same seven candidate models across the six core performance metrics.

Among seven evaluated representative architectures, ConvNeXt-Tiny consistently outperformed all baselines across binary and six-class diagnostic tasks on the according internal test set. In the six-class subtyping task, it performed well across most metrics (Table S7), especially achieving the highest accuracy of 83.4% (95% CI: 79.6–87.0%) and a macro-AUC of 0.968 (Figure 1B). Notably, although other architectures such as Swin-Tiny also performed better on the six-class subtyping, ConvNeXt-Tiny was able to accurately differentiated visually ambiguous subtypes (including cup ear and cryptotia) with an accuracy rate of 82.1%. In addition, this robust discriminative power extended to the binary anomaly screening task, where the model ranked first in each metric (Table S8), with an accuracy of 88.2% and an AUC of 0.949 (Figure 1A).

To further elucidate the mechanisms underlying ConvNeXt-Tiny’s superior performance, we retrospectively analyzed the training dynamics (Figure S2). As visually evident, convolutional architectures demonstrated faster convergence and stronger resistance to late-stage overfitting compared to pure Transformer models (e.g., ViT-Base). Given its optimal balance of peak classification accuracy, rapid convergence, and stability, ConvNeXt-Tiny was selected as the primary backbone for all subsequent evaluations.

### 3.2. Model Performance in Anomaly Screening and Deformity Subtyping

**Figure 2:**
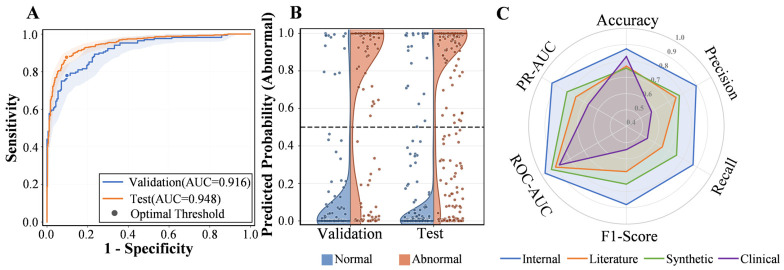
Comprehensive performance evaluation of the binary abnormality screening and six-class deformity subtype identification models. (**A**) Receiver Operating Characteristic (ROC) curves for the binary abnormality screening model. This panel displays the performance on the Validation set_2class and Internal test set_2class. (**B**) The distribution of probabilities for normal and abnormal samples predicted by the binary model. It illustrates the predicted probability of samples being classified as “Abnormal” across the validation and test sets. Blue and orange denote samples with true “Normal” and “Abnormal” labels, respectively, and the black dashed line (0.5) represents the default classification threshold, reflecting the model’s discriminative ability. (**C**) A radar chart of the six-class deformity subtype identification model’s performance across four different evaluation cohorts. It compares the model’s performance on the Internal, Literature, Synthetic, and Clinical datasets across six metrics.

In the binary anomaly screening task, ConvNeXt-Tiny achieved high diagnostic efficacy. Evaluated on the Internal test set_2class, the model achieved an overall accuracy of 88.2% (95% CI: 85.8–90.2%), accompanied by a ROC-AUC of 0.949 and a PR-AUC of 0.950 (Figure 2A). From a clinical translation perspective, the model prioritized a high specificity of 93.5% (identifying normal auricles) and maintaining a reliable sensitivity of 82.4%. Notably, the model exhibited a low false positive rate of 6.5% (30 misclassifications), underscoring its robust capacity to accurately recognize healthy neonates. Detailed classification results and the distribution of model classification are provided in Figure 2B and Figure S3. However, to minimize clinically consequential false negatives, we optimized a screening-oriented operating point, successfully elevating sensitivity to 90.26% (specificity: 86.06%). Adjusted for a realistic population prevalence (57.46%), the expected PPV and NPV remained robust at 89.74% and 86.74%, respectively (Supplementary Table S9). Additionally, Decision Curve Analysis and calibration curves confirmed the model’s significant clinical net benefit and reliable risk prediction capabilities (Supplementary Figures S4 and S5).

After fine-tuning the best backbone architecture and optimizing the weights for the dataset, we systematically evaluated six-class subtyping performance across four distinct cohorts (Figure 2C, Tables S10A and S11). On the Internal test set, which reflects the final optimized model development performance, the model demonstrated robust discriminative capacity, yielding an accuracy of 87.4% and a macro-AUC of 0.976. Notably, it exhibited high sensitivity for cryptotia (92.9%) and microtia (90.2%). Evaluated on the Literature test set, the accuracy decreased to 76.8%. This performance degradation was primarily driven by markedly lower sensitivities for lop and Stahl’s ears, indicating that the heterogeneity of photographic angles and lighting conditions in non-standardized literature images significantly interfered with the recognition of these specific subtypes. Regarding the Synthetic stress test set, the model achieved an accuracy of 75.6%. Importantly, in the real-world prospective clinical cohort, the model maintained an accuracy of 82.7% (Table S10B), which was predominantly driven by the high prevalence of the cup/constricted ear phenotype (*n* = 55) combined with the model’s high sensitivity (94.5%) for this specific class. Overall, the evaluations validated the model across multiple scenarios. It delivers precise classifications on internal data and adapts successfully to independent external cohorts. Importantly, it remains robust against rare subtypes under controlled stress testing. Furthermore, the stratified 5-fold cross-validation showed that the model achieved highly consistent metrics across all folds (mean accuracy: 84.31% ± 0.84%; macro-F1: 83.26% ± 1.11%; ROC-AUC: 0.9709 ± 0.0036), which suggests the robustness of the optimal ConvNeXt-Tiny model and its good generalization independent of data partitioning (Table S12).

### 3.3. Latent Feature Space Visualization and Misclassification Analysis

**Figure 3:**
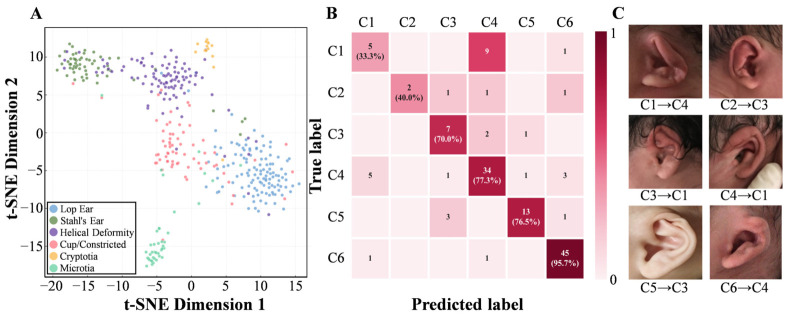
Feature space distribution and diagnostic performance analysis of the six-class model. (**A**) t-SNE visualization of the six-class deformity feature space. Scatter plot illustrating the 2D embedding of high-dimensional features extracted by the ConvNeXt-Tiny model via t-SNE (perplexity = 30). Colors correspond to the six deformity subtypes. The separation and overlap of clusters reflect the model’s discriminative power and morphological similarity representation. (**B**) Normalized confusion matrix for the literature test set. Rows and columns denote true and predicted classes, respectively (C1–C6: Lop Ear, Stahl’s Ear, Helical Deformity, Cup/Constricted Ear, Cryptotia, and Microtia). Diagonal cells indicate the counts and percentages of correct classifications, with the color scale representing normalized proportions. (**C**) Representative misclassified clinical cases. Annotations indicate the error flow (True class → Predicted class), illustrating typical confusion between morphologically similar subtypes and providing clinical insights into model errors.

t-SNE visualization of the deep features (Figure 3A) demonstrated that the six deformity subtypes formed distinct clusters. Notably, microtia and cryptotia were positioned at the distal ends of the latent space with substantial margins of separation, indicating their high morphological distinctiveness in deep representation. Conversely, the clusters for lop ear and cup/constricted ear were relatively adjacent but maintained a discernible inter-class boundary, consistent with their shared morphological alterations in the superior helix. A similar spatial proximity was observed between Stahl’s ear and helical deformity. These observations confirm that ConvNeXt-Tiny effectively encodes the intrinsic morphological divergence of the subtypes in its deep feature space.

Independent analysis of the evaluation cohorts revealed that while the model performed highly accurately on the internal baseline, unstandardized photographic conditions in the literature test set (Figure 3B) exacerbated morphological boundary confusions. Specifically, this cohort revealed prominent bidirectional misclassifications between lop and cup/constricted ears (*n* = 9 and *n* = 5, respectively). Similar confusions between adjacent subtypes (such as Stahl’s ear and helical deformity) were also observed across the individual sets. An aggregated matrix of all 811 instances alongside other individual matrices was provided in Figure S6. In addition, a retrospective visual review of these high-frequency error cases (Figure 3C) revealed that a substantial proportion of these auricles exhibited composite morphological features-concurrently displaying structural traits of multiple subtypes (Text S1). In such ambiguous borderline instances, the model tended to output a prediction aligned with the visually dominant deformity trait.

### 3.4. Visual Interpretability and Spatial Localization of Morphological Features

**Figure 4:**
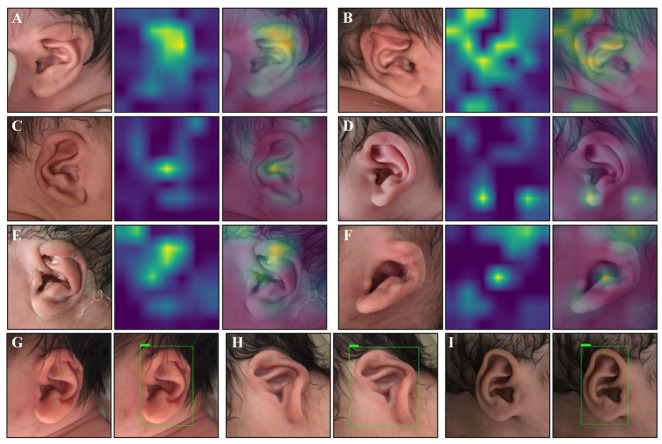
Visual interpretability and Region-of-Interest (ROI) localization of the diagnostic system. (**A**–**F**) Feature activation maps for the six ear deformity subtypes. Each panel sequentially displays the original input (**left**), activation heatmap (**middle**), and fused overlay (**right**). Warm-colored regions indicate the core morphological features driving the classification decisions. (**G**–**I**) Ear object detection based on the YOLO architecture. Green bounding boxes mark the final localized ROIs against complex backgrounds.

The automated region-of-interest (ROI) extraction using the YOLOv11L detector achieved a mean Average Precision (mAP@0.5) of 0.995 and a stringent mAP@0.5:0.95 of 0.964. In addition, the model demonstrated highly stable convergence during training, achieving a final validation classification (CLS) loss of 0.136 and a distribution focal loss (DFL) of 1.059, which showed that the YOLOv11L detector could effectively help isolate the target auricle and eliminate background interference (Figure 4G–I). To further isolate the effect of YOLO-based cropping on classification performance, we conducted ablation experiments in which the classification model was evaluated with and without this preprocessing step. The results revealed no substantial difference between the two settings across all four datasets (Table S3). Furthermore, we utilized Grad-CAM to explore the spatial distribution of the ConvNeXt-Tiny classifier’s attention. As illustrated in Figure 4A–F, the generated heatmaps suggest that the model’s focus frequently aligns with clinically relevant morphological regions. The network visually highlighted areas corresponding to disease-specific pathological features across all subtypes (such as the abnormal third crus in Stahl’s ears and the underdeveloped framework in microtia), indicating a strong spatial concordance with clinical diagnostic intuition.

### 3.5. Quantitative Evaluation of Therapeutic Efficacy via Similarity-Based Severity Score

**Figure 5:**
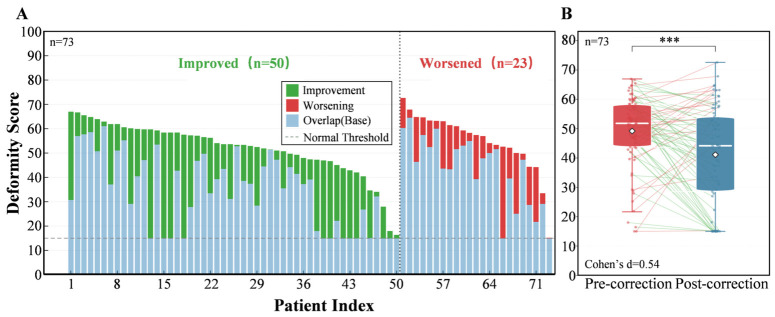
Quantitative assessment of neonatal ear deformities pre and post correction. (**A**) A stacked bar chart detailing deformity score changes for 73 paired ears. Green and red segments denote the improved (*n* = 50) and worsened (*n* = 23) cohorts, respectively. The dashed line represents the classification threshold for normal morphology (score = 15). (**B**) A paired boxplot with overlaid scatter plot illustrating the distribution of pre- and post-correction deformity scores. Color-coded lines connect paired observations from the same patient, tracking individual trajectories (faint green for improvement, and faint red for worsening). “***” indicates that the difference of deformity score between pre and post correction is extremely significant.

To validate the sensitivity of the severity scoring system in capturing longitudinal morphological trajectories, we evaluated paired pre- and post-treatment images from 73 clinical cases. The mean severity scores significantly decreased from 49.2 ± 12.8 before treatment to 41.1 ± 17.0 following the intervention, reflecting a mean reduction of 8.1 points (Wilcoxon signed-rank test, *p* = 0.0004, *Cohen’s d* = 0.54) (Figure 5B). Individual patient trajectories (Figure 5A) visually corroborated this trend, demonstrating a consistent shift toward the lower-severity spectrum post treatment. Overall, these quantitative metrics confirm that the proposed scoring framework effectively captures the overall therapeutic response, yielding a statistically significant reduction in deformity severity.

To visually illustrate this concordance, several representative clinical trajectories are showcased in Supplementary Figure S7. The computed severity scores accurately mapped to clinical observations across varying degrees of recovery. Specifically, the scores robustly reflected complete morphological normalization (Figure S7A–C), partial regression of specific abnormalities (Figure S7F), and persisting residual deformities (Figure S7D,E). Collectively, these cases demonstrate that our severity scores are highly consistent with human-perceived improvements, providing a reliable quantitative proxy for treatment efficacy.

### 3.6. Evaluation of the Prototype Diagnostic Workstation

To demonstrate the technical feasibility of real-world application, we deployed an end-to-end prototype web-based diagnostic workstation (https://earcad.zcmu.edu.cn/ear/, accessed on 21 June 2026, Figure S8). Upon image upload, the system executes a fully automated, cascaded inference pipeline: a YOLOv11L model standardizes the auricular region of interest, followed by the optimized ConvNeXt-Tiny backbone, which performs parallel binary screening and fine-grained subtyping. Concurrently, a dedicated metric learning module computes a continuous deformity severity score. To ensure algorithmic transparency and foster clinical trust, diagnostic outputs are rendered alongside Grad-CAM heatmaps, providing interpretable morphological evidence for the model’s predictions. In preliminary performance benchmarks, the prototype demonstrated high efficiency. The average processing latency is approximately 1 to 2s per image for the core classification and scoring modules, with an additional 2s required when the YOLO extraction module is explicitly activated. For batch analysis, the total processing time scales linearly, successfully showcasing the technical capability to provide near real-time diagnostic support and automated reporting.

## 4. Discussion

In primary healthcare settings, the clinical management of neonatal auricular deformities encompasses several related but distinct stages: initial screening, precise diagnosis (subtyping), baseline severity grading, and longitudinal treatment monitoring [7]. To address the critical gaps across this continuum, we developed and validated a comprehensive intelligent system. Our empirical evaluations demonstrated that this system effectively maps to these distinct clinical tasks. First, it achieves high accuracy in the binary screening of anomalies and the fine-grained diagnostic classification of specific subtypes. Furthermore, by introducing a continuous anomaly score, the model introduces the quantitative assessment of baseline severity and the precise tracking of post-treatment morphological improvements. By shifting from static categorical classification to dynamic severity quantification, this work overcomes the inherent limitations of subjective visual appraisal, offering an objective, standardized, and interpretable paradigm for longitudinal treatment monitoring.

### 4.1. Architectural Superiority and Strategic Backbone Selection

Unlike existing single-stage diagnostic models [18], our system introduces critical optimizations in both architectural design and backbone selection. First, we innovatively applied a cascade strategy: initial binary screening followed by fine-grained subtyping. Since normal cases dominate real-world screening, filtering them out early prevents healthy samples from triggering computationally expensive downstream classifications, thereby meeting the low-overhead deployment demands of primary care settings. Second, our comparative analyses identified ConvNeXt-Tiny as the optimal backbone; unlike pure Transformers (e.g., ViT) that often overfit small-to-medium medical datasets, ConvNeXt balances the optimization stability of traditional CNNs with the representational capacity of modern Transformers [22]. The integration of this cascaded workflow with the selected backbone not only yields high accuracy in capturing subtle anatomical variations but also provides a practical foundation for future large-scale clinical translation.

### 4.2. Domain Generalization and Real-World Challenges

At the data curation level, this study augmented the dataset by systematically retrieving images from peer-reviewed studies. This strategy successfully integrated cryptotia (a clinically significant subtype previously overlooked by Ren et al. [18]) into the classification taxonomy. This integration establishes a comprehensive six-class diagnostic standard that accurately reflects the true clinical spectrum [8,23]. Nevertheless, the model exhibited performance degradation on the Literature test set, highlighting how non-standardized factors (such as varied photographic angles, illumination, backgrounds, and image clarity) interfere with robust deep feature extraction. This study validated the feasibility of employing Denoising Diffusion Probabilistic Models (DDPMs) to generate high-fidelity synthetic images as a stress test for evaluating model robustness. This approach provided insight into model performance on a balanced cohort of rare subtypes without claiming external validation. It is important to emphasize that synthetic images cannot fully replicate clinical realism or substitute for real-world heterogeneity. Although these data facilitate robust stress testing, there is a real risk of overfitting the model to the original training distributions and thus needs to be interpreted with caution. Furthermore, establishing a standardized image acquisition protocol is a key prerequisite for multi-center clinical translation.

Furthermore, we addressed the specific risk of data leakage. A major concern is that left and right ears from the same neonate might be split into different data partitions. To resolve this, we conducted a rigorous ablation study on the well-annotated BabyEar4K cohort. A strict patient-level split (Table S13) demonstrated negligible performance differences compared to random image-level splitting. This empirical evidence confirms that the simultaneous presence of bilateral ears across partitions does not artificially inflate performance, as the model primarily captures generalizable morphological structures rather than patient-specific photographic artifacts. While this patient-level analysis reduces concerns about bilateral ear leakage, it does not fully replace the need for a locked, multi-center external validation for the full six-class task. For the literature-derived dataset, we inherently prevented leakage risks during the data collection phase. We used a strict screening protocol to avoid extracting too many images from a single publication. We also performed rigorous cross-referencing, image deduplication, and excluded low-quality photographs. These procedures fundamentally eliminated the possibility of duplicate or highly correlated samples appearing across different partitions.

### 4.3. Clinical Translation of the Severity Score

Another clinical contribution of this study is the objective quantification of morphological severity. Prior research has predominantly relied on the prediction confidence of classification networks as a proxy metric for deformity severity [16,19]. However, because these models do not explicitly learn severity as a continuous variable, their output probabilities lack reliable clinical calibration. To circumvent this limitation, we introduced a supervised contrastive learning framework to extract the deep feature prototype of the “normal auricle” as a baseline. By computing the cosine similarity between the latent representation of the query sample and this reference prototype, macroscopic morphological deviations are directly mapped into a continuous deformity severity score. This approach eliminates the need for labor-intensive, fine-grained severity annotations while demonstrating high sensitivity to therapeutic changes in real-world longitudinal cohorts. Crucially, this continuous index establishes an objective, longitudinal assessment metric for neonatal non-invasive ear molding, thereby overcoming the subjective limitations inherent in traditional visual appraisals. Ultimately, it has the potential to empower clinicians to quantitatively monitor treatment trajectories and support data-driven clinical decisions, such as determining the optimal endpoint for ear molding therapy. However, we observed an unexpected increase in anomaly scores post treatment for 23 paired cases. Analysis revealed that this score inversion was primarily driven by inconsistent photographic conditions across time points (such as severe variations in camera angle, lighting, and image scale), which confounded the similarity algorithm. Furthermore, in a smaller subset, these increased scores reflected true clinical non-responders or partial improvements, where persistent minor structural anomalies disproportionately influenced the algorithm’s assessment.

### 4.4. The Dilemma of Composite Deformities and Single-Label Boundaries

Retrospective analysis reveals that misclassifications are concentrated in subtypes with ambiguous boundaries (e.g., bidirectional confounding between Stahl’s and helical deformities; see details in Text S1). This primarily stems from the clinical reality of “composite deformities”, where features of multiple subtypes coexist. However, the hard-label paradigms force a single dominant category, violating morphological co-occurrence and causing localized overfitting. Consequently, future intelligent diagnostic frameworks must evolve toward multi-label classification networks or detection architectures augmented by anatomical key points.

From a clinical perspective, these boundary misclassifications carry varying risks. Confusions among mild, non-surgical deformities (e.g., lop, Stahl’s, helical) are clinically tolerable due to their shared non-invasive molding pathways. Conversely, missing cryptotia or misclassifying microtia as a milder subtype are clinically dangerous, potentially delaying time-sensitive surgical referrals or early interventions [11]. Given these risks, clinicians facing ambiguous cases should not rely solely on hard labels, but integrate Grad-CAM heatmaps, continuous severity scores, and clinical judgment to ensure optimal patient triage.

### 4.5. Limitations and Future Directions

While this intelligent workstation shows substantial promise, several limitations warrant consideration. First, the model was predominantly trained on single-center data, with specific subtypes like cryptotia remaining relatively rare [24,25], which may lead to a classification bias and limit the diagnostic sensitivity in real-world clinical settings. The system showed promising preliminary performance in a small prospective clinical cohort, but validation for rare subtypes remains underpowered, necessitating future large-scale, multi-center trials to rigorously confirm efficacy for minority classes. Furthermore, while the DDPM-generated set served as a valuable stress test, these synthetic images may inadvertently resemble training distributions and cannot fully substitute for true real-world heterogeneity. Second, relying exclusively on 2D images without multi-dimensional clinical metadata (e.g., postnatal age, parental preferences [11]) restricts the platform from autonomously prescribing specific clinical actions [26]. To overcome this problem, future studies could enhance diagnostic robustness in complex clinical scenarios by integrating recent methodological advances [27,28] in multimodal image segmentation. Third, the heuristic piecewise severity scoring system, although effective in capturing longitudinal shifts, currently functions primarily as a learned morphological similarity index rather than a fully validated clinical scale. Moving forward, future prospective trials must rigorously calibrate this score against established metrics by correlating it with blinded expert clinician ratings and objective anatomical morphometric measurements. Moreover, it is imperative to quantitatively validate the interpretability heatmaps in future studies. We plan to employ specialists to rate heatmap plausibility and quantify localization overlap against manually annotated anatomical landmarks. These comprehensive validation steps, alongside large-scale, multi-center trials for regulatory compliance and system optimization for edge devices, are essential to ensure safe and effective deployment in primary care.

## 5. Conclusions

In conclusion, this study establishes an interpretable deep learning framework centered on a supervised contrastive learning-based severity scoring system for the precise screening, subtyping, and longitudinal monitoring of neonatal auricular deformities. By coupling a cascaded classification pipeline with a similarity-based severity score, we provide a robust solution to the long-standing challenges of diagnostic subjectivity and the absence of quantitative tracking metrics. Evaluated across multiple distinct cohorts and a controlled synthetic stress test, the system demonstrates high diagnostic accuracy and significant responsiveness to therapeutic interventions. This intelligent paradigm has the potential to standardize the management of neonatal auricular anomalies, facilitating early intervention and optimizing clinical outcomes in primary healthcare settings via a web-based clinical service.

## Figures and Tables

**Table 1:** Sample distribution of the multi-source neonatal ear dataset.

Category	Source				Total
	Public	Literature	Synthetic	Clinical	
Normal	2136	0	0	0	2136
Lop ear	658	69	30	4	761
Stahl’s ear	264	27	30	5	326
Helical deformity	463	50	30	5	548
Cup/Constricted ear	154	218	30	55	457
Cryptotia	1	88	30	3	122
Microtia	19	236	30	9	294
Abnormal	1559	688	180	81	2508
Total	3695	688	180	81	4644

Note: The table summarizes the composition of the multi-source neonatal ear dataset. The public subset was sourced from the BabyEar4K open-source repository.

## Data Availability

The datasets generated and analyzed during the current study, comprising fully de-identified images approved for public sharing, are publicly available in the Zenodo repository (https://doi.org/10.5281/zenodo.20416251). Furthermore, to ensure complete methodological reproducibility, the full source codes, including preprocessing scripts, split configurations, and trained weights, are accessible on both Zenodo (https://doi.org/10.5281/zenodo.20414922) and GitHub (https://github.com/AudioGen06/EarCAD, accessed on 21 June 2026).

## Supplementary Materials

The following supporting information can be downloaded at: https://www.mdpi.com/article/10.3390/biology15130985/s1, Table S1: Training implementation details for the DDPM; Table S2: Dataset partitioning and sample distribution for the binary and six-class diagnostic tasks; Table S3: Ablation study on the impact of YOLOv11 ROI extraction across the four distinct evaluation cohorts; Table S4: Training implementation details for the classification models; Table S5: Training details of the deformity severity scoring; Table S6: Similarity Score Distribution in Normal and Malformed Ears; Table S7: Detailed performance metrics of all evaluated deep learning models for the six-class subtype identification on the internal test set; Table S8: Detailed performance metrics of all evaluated deep learning models for the binary anomaly screening on the internal test set; Table S9: Comprehensive performance metrics of the binary screening model across multiple decision thresholds and at the screening-oriented operating point; Table S10: Comprehensive performance metrics of the six-class deformity subtype identification model (A) Overall performance across four evaluation cohorts (B) Class-specific performance on the prospective clinical test set (*n* = 81); Table S11: Per class PR AUC across evaluation cohorts; Table S12: Detailed 5-fold cross-validation performance metrics of the optimal model evaluated on the internal test set; Table S13: Performance comparison of the ConvNeXt-Tiny model on the binary screening task using Image-level vs. Patient-level data splitting strategies; Figure S1: Representative failure cases of synthetic images generated by the Denoising Diffusion Probabilistic Model; Figure S2: Training dynamics of different deep learning backbones in the intelligent diagnosis task of neonatal ear deformities; Figure S3: Normalized confusion matrix of the binary classification task (normal vs. deformed) on the test set; Figure S4: Clinical utility and probability calibration of the binary screening model; Figure S5: Overall calibration curve for the six-class deformity subtype identification model; Figure S6: Confusion matrices for the aggregated and individual test sets; Figure S7: Clinical tracking of neonatal ear deformities pre- and post-treatment; Figure S8: The web-based intelligent auxiliary diagnosis system interface; Text S1: Anatomical Basis for Systematic Misclassifications.
