# Supplementary material for: An Interpretable Deep Learning System for Fine-Grained Classification and Longitudinal Tracking of Neonatal Auricular Deformities
Source: Biology (Basel). 2026 Jun 23;15(13):985. doi: 10.3390/biology15130985 (PMC13360241; doi:10.3390/biology15130985)
Supplement: Supplementary file 1 [file biology-15-00985-s001.zip › biology-4367043-supplementary.pdf]

An Interpretable Deep Learning System for Fine-Grained Classification and Longitudinal Tracking of Neonatal Auricular Deformities

Yihui Feng[1,†], Xujun Hu[1,†], Xiwen Zhang[1], Xiaobao Ma[2], Jialin Xie[3], Jianyong Chen[2,*], Yangyang Yuan[1,*]

[1] School of Medical Technology and Information Engineering, Zhejiang Chinese Medical University, Hangzhou, 310053, China

[2] Department of Otorhinolaryngology-Head and Neck Surgery, Xinhua Hospital, Shanghai Jiaotong University School of Medicine, Shanghai, 200092, China

[3] The Second School of Clinical Medicine, Zhejiang Chinese Medical University, Hangzhou, 310053, China

[*] Authors to whom correspondence should be addressed.

[†] These authors contributed equally to this work.

Table S1. Training implementation details for the DDPM.

Parameter	Value
Backbone architecture	Conditional U-Net (UNet2DConditionModel)
Downsampling blocks	4
Upsampling blocks	4
Channel dimensions	64, 128, 256, 256
Cross-attention dimension	256
Input resolution	128 × 128 pixels
Optimizer	AdamW
Initial learning rate	$1{\times}10^{-4}$
Learning rate scheduler	Cosine annealing with 500-step linear warmup
Batch size	8
Gradient accumulation steps	2
Training epochs	200
Mixed precision	FP16
Diffusion timesteps (training)	1,000
Diffusion timesteps (inference)	250
Beta schedule	Squaredcos_cap_v2
Implementation framework	PyTorch + HuggingFace Diffusers

Note: DDPM, denoising diffusion probabilistic model. All hyperparameters were used for the training procedure described in Section 2.2 of the main text.

Table S2. Dataset partitioning and sample distribution for the binary and six-class diagnostic tasks.

Dataset	Binary classification			Six-class classification						
	Normal	Abnormal	Total	Lop	Stahl	Helical	Cup	Cryptotia	Microtia	Total
Training set	1,706	1,797	3,503	575	229	404	264	58	167	**1,697**
Internal test set	430	450	880	137	57	99	64	14	41	**412**
Literature test set	--	--	--	15	5	10	44	17	47	**138**
Synthetic stress test set	--	--	--	30	30	30	30	30	30	**180**
Clinical test set	--	--	--	4	5	5	55	3	9	**81**
**Total**	**2,136**	**2,247**	**4,383**	**761**	**326**	**548**	**457**	**122**	**294**	**2,508**

Note: The table details the sample allocation for abnormality screening (binary classification) and deformity subtype identification (six class classification) across the training set, three independent real world test sets, and one controlled synthetic stress test. Dashes (--) indicate that the dataset was not utilized for the corresponding evaluation task. Abbreviations for the six-class deformity subtypes are as follows: Lop, Lop ear; Stahl, Stahl's ear; Helical, Helical deformity; Cup, Cup/Constricted ear.

Table S3. Ablation study on the impact of YOLOv11 ROI extraction across the four distinct evaluation cohorts.

Dataset	Configuration	Accuracy	Precision (Macro)	Recall (Macro)	F1-Score (Macro)	ROC-AUC (Macro)	PR-AUC（Macro）
**Internal test set**	Without YOLO	0.8738 (0.8398 - 0.9029)	0.8945 (0.8575 - 0.9277)	0.8726 (0.8334 - 0.9103)	0.8806 (0.8444 - 0.9120)	0.9762 (0.9662 - 0.9848)	0.9272 (0.9023 - 0.9523)
	With YOLO	0.8665 (0.8325 - 0.8981)	0.8902 (0.8612 - 0.9184)	0.8614 (0.8163 - 0.9001)	0.8726 (0.8331 - 0.9063)	0.9756 (0.9656 - 0.9835)	0.9228 (0.8956 - 0.9497)
**Literature test set**	Without YOLO	0.7681 (0.6957 - 0.8333)	0.7517 (0.5532 - 0.8296)	0.6547 (0.5470 - 0.7629)	0.6784 (0.5537 - 0.7795)	0.9021 (0.8271 - 0.9543)	0.7584 (0.6446 - 0.8523)
	With YOLO	0.7536 (0.6812 - 0.8188)	0.7710 (0.5728 - 0.8419)	0.6420 (0.5340 - 0.7609)	0.6735 (0.5371 - 0.7772)	0.8986 (0.8267 - 0.9490)	0.7559 (0.6474 - 0.8453)
**Synthetic stress test set**	Without YOLO	0.7556 (0.6944 - 0.8167)	0.7756 (0.7210 - 0.8318)	0.7556 (0.6935 - 0.8157)	0.7553 (0.6875 - 0.8139)	0.9313 (0.9047 - 0.9570)	0.8206 (0.7656 - 0.8828)
	With YOLO	0.7222 (0.6556 - 0.7833)	0.7488 (0.6892 - 0.8082)	0.7222 (0.6592 - 0.7853)	0.7202 (0.6429 - 0.7804)	0.9239 (0.8945 - 0.9503)	0.8022 (0.7455 - 0.8633)
**Clinical test set**	Without YOLO	0.8272 (0.7531 - 0.9012)	0.5778 (0.4057 - 0.7155)	0.5492 (0.4147 - 0.6639)	0.5436 (0.3911 - 0.6484)	0.8747 (0.7746 - 0.9436)	0.6688 (0.5070 - 0.8371)
	With YOLO	0.8272 (0.7407 - 0.9012)	0.5778 (0.3952 - 0.7238)	0.5492 (0.4307 - 0.6640)	0.5436 (0.3950 - 0.6595)	0.8722 (0.7782 - 0.9412)	0.6627 (0.5101 - 0.8395)

Note: Data are presented as value with 95% Confidence Interval obtained via 1,000 bootstrap iterations.

Table S4. Training implementation details for the classification models.

Parameter	Value
**Candidate architectures**	ConvNeXt-Tiny,ResNet-50,EfficientNet-B3,MobileNetV3-Large, DenseNet-121, Swin Transformer-Tiny, ViT-Base
**Pre-training**	ImageNet-1K weights
**Input resolution**	224 × 224 pixels (cropped and resized)
**Optimizer**	Adam
**Initial learning rate**	1×10^{-4}
**Learning rate scheduler**	ReduceLROnPlateau (decay factor = 0.5, patience = 3 epochs)
**Batch size**	32
**Loss function**	Weighted Cross-Entropy Loss
**Training epochs**	30 (with early stopping)
**In-training validation split**	10% dynamic subset
**Implementation framework**	PyTorch

Note: All hyperparameters were used for the training procedure described in Section 2.5 of the main text.

Table S5. Training details of the deformity severity scoring.

Parameter / Component	Specification
**Encoder Architecture**	Pre-trained ResNet-50 (fc layer removed)
**Projection Head**	Linear (2048 to 512), BatchNorm1d, ReLU, Linear (512 to 128), L2 Normalization
**Contrastive Labels**	7-class (1 normal class and 6 specific deformity subtype classes)
**Loss Function**	Supervised Contrastive Loss (temperature = 0.1)
**Optimizer**	Adam (learning rate = 1e-4, weight decay = 1e-4)
**Learning Rate Schedule**	Cosine Annealing (T_max = 50)
**Training Epochs**	30
**Batch Size**	32
**Data Augmentations**	RandomResizedCrop (224x224, scale 0.8-1.0), RandomHorizontalFlip (p=0.3), RandomRotation (10°), ColorJitter (0.2, 0.2, 0.2, 0.1), RandomGrayscale (p=0.2)
**Prototype Construction**	Computed exclusively on normal samples from the held-out validation set (20% split)
**Evaluation Data**	Strictly evaluated on the held-out validation set (unseen during training)

Table S6. Similarity Score Distribution in Normal and Malformed Ears.

Category	Mean	Standard deviation	Minimum	Maximum	Median
**Normal**	85.60	3.36	71.67	91.61	86.14
**Abnormal**	74.39	7.72	57.36	88.88	75.37
**Lop ear**	76.61	4.16	68.68	87.87	76.15
**Stahl's ear**	77.45	6.57	65.28	88.23	77.55
**Helical deformity**	80.69	5.09	69.04	88.88	81.74
**Cup/Constricted ear**	71.15	6.97	62.28	86.29	67.88
**Cryptotia**	64.97	1.90	62.86	68.31	64.43
**Microtia**	61.67	2.33	57.36	67.04	61.45

Note: The similarity score (ranging from 0 to 100) reflects the cosine similarity between the deep feature vector of the input image and the normalized prototype center of normal auricles extracted via supervised contrastive learning. A higher score denotes a closer morphological resemblance to a healthy neonatal auricle.

Table S7. Detailed performance metrics of all evaluated deep learning models for the six-class subtype identification on the internal test set.

Model	Accuracy (95% CI)	Macro-Precision (95% CI)	Macro-Recall (95% CI)	Macro-F1 (95% CI)	ROC-AUC (95% CI)	PR-AUC (95% CI)
**ConvNeXt-Tiny**	0.8341 (0.7962 – 0.8697)	0.8471 (0.7998 – 0.8855)	0.8168 (0.7635 – 0.8634)	0.8297 (0.7818 – 0.8671)	0.9683 (0.9567 – 0.9784)	0.8890 (0.8498 – 0.9247)
**Swin Transformer Tiny**	0.8246 (0.7820 – 0.8602)	0.8266 (0.7744 – 0.8739)	0.8039 (0.7538 – 0.8537)	0.8130 (0.7605 – 0.8564)	0.9695 (0.9595 – 0.9785)	0.8852 (0.8470 – 0.9220)
**ResNet-50**	0.7962 (0.7583 – 0.8341)	0.8037 (0.7491 – 0.8513)	0.7803 (0.7248 – 0.8267)	0.7863 (0.7316 – 0.8322)	0.9637 (0.9512 – 0.9751)	0.8649 (0.8207 – 0.9033)
**EfficientNet-B3**	0.7915 (0.7512 – 0.8294)	0.7602 (0.7039 – 0.8156)	0.7677 (0.7146 – 0.8213)	0.7602 (0.6995 – 0.8088)	0.9534 (0.9332 – 0.9687)	0.8446 (0.7974 – 0.8868)
**DenseNet-121**	0.7820 (0.7440 – 0.8246)	0.8054 (0.7500 – 0.8519)	0.7513 (0.6950 – 0.8062)	0.7725 (0.7179 – 0.8174)	0.9479 (0.9322 – 0.9631)	0.8239 (0.7768 – 0.8764)
**Vit-Base**	0.7796 (0.7370 – 0.8152)	0.7822 (0.7283 – 0.8320)	0.7656 (0.7145 – 0.8132)	0.7706 (0.7186 – 0.8094)	0.9592 (0.9457 – 0.9710)	0.8489 (0.8067 – 0.8913)
**MobileNetV3-Large**	0.7654 (0.7251 – 0.8033)	0.7782 (0.7300 – 0.8197)	0.7582 (0.7056 – 0.8074)	0.7662 (0.7155 – 0.8083)	0.9522 (0.9379 – 0.9646)	0.8393 (0.7940 – 0.8815)

Note: Data are presented as value with 95% Confidence Interval obtained via 1,000 bootstrap iterations.

Table S8. Detailed performance metrics of all evaluated deep learning models for the binary anomaly screening on the internal test set.

Model	Accuracy (95% CI)	Macro-Precision (95% CI)	Macro-Recall (95% CI)	Macro-F1 (95% CI)	ROC-AUC (95% CI)	PR-AUC (95% CI)
**ConvNeXt-Tiny**	0.8818 (0.8580–0.9023)	0.8867 (0.8674–0.9055)	0.8794 (0.8578–0.9000)	0.8808 (0.8578–0.9007)	0.8794 (0.8574–0.9010)	0.9493 (0.9350–0.9630)
**Swin Transformer Tiny**	0.8636 (0.8409–0.8852)	0.8689 (0.8479–0.8903)	0.8610 (0.8391–0.8838)	0.8623 (0.8381–0.8855)	0.8610 (0.8363–0.8821)	0.9405 (0.9240–0.9546)
**ResNet-50**	0.8625 (0.8386–0.8864)	0.8725 (0.8513–0.8933)	0.8589 (0.8378–0.8794)	0.8605 (0.8359–0.8848)	0.8589 (0.8370–0.8816)	0.9417 (0.9261–0.9564)
**EfficientNet-B3**	0.8625 (0.8398–0.8864)	0.8708 (0.8500–0.8925)	0.8592 (0.8354–0.8808)	0.8607 (0.8377–0.8851)	0.8592 (0.8367–0.8826)	0.9342 (0.9172–0.9501)
**DenseNet-121**	0.8602 (0.8386–0.8841)	0.8707 (0.8490–0.8913)	0.8566 (0.8349–0.8783)	0.8581 (0.8342–0.8808)	0.8566 (0.8333–0.8784)	0.9345 (0.9165–0.9508)
**Vit-Base**	0.8511 (0.8284–0.8739)	0.8563 (0.8326–0.8782)	0.8484 (0.8252–0.8719)	0.8497 (0.8252–0.8729)	0.8484 (0.8237–0.8720)	0.9277 (0.9090–0.9434)
**MobileNetV3-Large**	0.8295 (0.8057–0.8534)	0.8361 (0.8121–0.8607)	0.8264 (0.8005–0.8500)	0.8275 (0.8016–0.8537)	0.8264 (0.8028–0.8495)	0.9204 (0.9018–0.9377)

Note: Data are presented as value with 95% Confidence Interval obtained via 1,000 bootstrap iterations.

Table S9. Comprehensive performance metrics of the binary screening model across multiple decision thresholds and at the screening-oriented operating point.

Decision Threshold	Sensitivity	Specificity	PPV (Test Set)	NPV (Test Set)
**0.1**	0.8599	0.9129	0.9005	0.8766
**0.2**	0.8361	0.9259	0.9119	0.8603
**0.3**	0.8171	0.9303	0.9149	0.8472
**0.4**	0.8052	0.9434	0.9288	0.8408
**0.5 (Default)**	0.7886	0.9477	0.9326	0.8302
**0.6**	0.7720	0.9521	0.9366	0.8199
**0.7**	0.7672	0.9564	0.9417	0.8175
**0.8**	0.7577	0.9608	0.9466	0.8122
**0.9**	0.7363	0.9651	0.9509	0.7996
**0.010 (High-Sensitivity)**	0.9026	0.8606	0.8559	0.9060

Note: The high-sensitivity threshold (0.010) represents a screening-oriented operating point optimized to achieve ≥90% sensitivity, minimizing false negatives (missed diagnoses). Adjusted for a realistic population prevalence of 57.46%, the expected Positive Predictive Value (PPV) and Negative Predictive Value (NPV) at this high-sensitivity point are 89.74% and 86.74%, respectively. The confusion matrix at this threshold is True Negatives (TN): 395, False Positives (FP): 64, False Negatives (FN): 41, and True Positives (TP): 380.

Table S10. Comprehensive performance metrics of the six-class deformity subtype identification model.

(A) Overall performance across four evaluation cohorts

Evaluation Cohort	Sample Size (n)	Accuracy **(95% CI)**	Macro-Precision **(95% CI)**	Macro-Recall **(95% CI)**	Macro-F1 **(95% CI)**	Macro-ROC-AUC **(95% CI)**
**Internal test set**	412	0.8738 (0.8398 - 0.9029)	0.8945 (0.8575 - 0.9277)	0.8726 (0.8334 - 0.9103)	0.8806 (0.8444 - 0.9120)	0.9762 (0.9662 - 0.9848)
**Literature test set**	138	0.7681 (0.6957 - 0.8333)	0.7517 (0.5532 - 0.8296)	0.6547 (0.5470 - 0.7629)	0.6784 (0.5537 - 0.7795)	0.9021 (0.8271 - 0.9543)
**Synthetic stress test set**	180	0.7556 (0.6944 - 0.8167)	0.7756 (0.7210 - 0.8318)	0.7556 (0.6935 - 0.8157)	0.7553 (0.6875 - 0.8139)	0.9313 (0.9047 - 0.9570)
**Clinical test set**	81	0.8272 (0.7531 - 0.9012)	0.5778 (0.4057 - 0.7155)	0.5492 (0.4147 - 0.6639)	0.5436 (0.3911 - 0.6484)	0.8747 (0.7746 - 0.9436)

(B) Class-specific performance on the prospective clinical test set (n=81)

**Deformity Subtype**	**Sample Size (n)**	**Precision (95% CI)**	**Recall / Sensitivity (95% CI)**	**Specificity (95% CI)**	**F1-Score (95% CI)**	**ROC-AUC (95% CI)**
**Lop ear**	4	1.0000 (0.0000 - 1.0000)	0.7500 (0.0000 - 1.0000)	1.0000 (1.0000 - 1.0000)	0.8571 (0.0000 - 1.0000)	0.8409 (0.5000 - 1.0000)
**Stahl’s ear**	5	1.0000 (0.0000 - 1.0000)	0.6000 (0.0000 - 1.0000)	1.0000 (1.0000 - 1.0000)	0.7500 (0.0000 - 1.0000)	0.9289 (0.8053 - 1.0000)
**Helical deformity**	5	0.0000 (0.0000 - 0.0000)	0.0000 (0.0000 - 0.0000)	1.0000 (1.0000 - 1.0000)	0.0000 (0.0000 - 0.0000)	0.6553 (0.3547 - 0.9134)
**Cup/Constricted ear**	55	0.8667 (0.7759 - 0.9395)	0.9455 (0.8772 - 1.0000)	0.6923 (0.5185 - 0.8669)	0.9043 (0.8440 - 0.9524)	0.8636 (0.7532 - 0.9566)
**Cryptotia (Rare)**	3	0.0000 (0.0000 - 0.0000)	0.0000 (0.0000 - 0.0000)	1.0000 (1.0000 - 1.0000)	0.0000 (0.0000 - 0.0000)	0.9872 (0.5000 - 1.0000)
**Microtia (Rare)**	9	0.6000 (0.3529 - 0.8462)	1.0000 (1.0000 - 1.0000)	0.9167 (0.8441 - 0.9718)	0.7500 (0.5000 - 0.9168)	0.9722 (0.9287 - 1.0000)

Note: Data are presented as values with 95% Confidence Intervals obtained via 1,000 bootstrap iterations. Panel A presents the macroscopic evaluation across cohorts, while Panel B details the fine-grained performance on the highly imbalanced clinical dataset to ensure transparency regarding rare subtypes.

Table S11. Per class PR AUC across evaluation cohorts.

**Deformity Subtype**	**Internal test set**	**Literature test set**	**Synthetic stress test set**	**Clinical test set**
**Lop ear**	0.9558 (0.9327 - 0.9789)	0.4148 (0.1945 - 0.6673)	0.8456 (0.7200 - 0.9436)	0.7067 (0.0208 - 1.0000)
**Stahl's ear**	0.9008 (0.8336 - 0.9576)	0.8087 (0.2351 - 1.0000)	0.8223 (0.6981 - 0.9320)	0.7050 (0.1180 - 1.0000)
**Helical deformity**	0.8591 (0.7825 - 0.9237)	0.7090 (0.3978 - 0.9472)	0.6678 (0.4825 - 0.8531)	0.2884 (0.0309 - 0.7255)
**Cup/Constricted ear**	0.8989 (0.8441 - 0.9449)	0.7821 (0.6582 - 0.8901)	0.7711 (0.6284 - 0.8948)	0.8793 (0.7679 - 0.9798)
**Cryptotia (Rare)**	0.9823 (0.9338 - 1.0000)	0.8589 (0.7007 - 0.9702)	0.9202 (0.8333 - 0.9866)	0.6389 (0.0000 - 1.0000)
**Microtia (Rare)**	0.9666 (0.9184 - 1.0000)	0.9766 (0.9446 - 0.9952)	0.8965 (0.8010 - 0.9649)	0.7945 (0.5235 - 0.9892)

Note: Data are presented as values with 95% Confidence Intervals obtained via 1,000 bootstrap iterations. PR AUC serves as an essential metric for evaluating predictive reliability on imbalanced minority classes.

Table S12. Detailed 5-fold cross-validation performance metrics of the optimal model evaluated on the internal test set.

Fold	Accuracy	Precision (Macro)	Recall (Macro)	F1 (Macro)	ROC-AUC	PR-AUC
**Fold 1**	83.65%	83.01%	80.85%	81.80%	0.9679	0.8537
**Fold 2**	83.92%	83.31%	82.47%	82.73%	0.9752	0.8912
**Fold 3**	85.56%	85.77%	84.06%	84.87%	0.9662	0.8780
**Fold 4**	83.38%	84.21%	81.57%	82.73%	0.9746	0.8946
**Fold 5**	85.01%	84.38%	84.16%	84.17%	0.9708	0.8865
**Mean ± SD**	**84.31% ± 0.84%**	**84.14% ± 0.97%**	**82.62% ± 1.32%**	**83.26% ± 1.11%**	**0.9709 ± 0.0036**	**0.8808 ± 0.0146**

Table S13. Performance comparison of the ConvNeXt-Tiny model on the binary screening task using Image-level vs. Patient-level data splitting strategies.

Splitting Strategy	Accuracy (95% CI)	Macro-Precision (95% CI)	Macro-Recall (95% CI)	Macro-F1 (95% CI)	ROC-AUC (95% CI)	PR-AUC (95% CI)
**Image-level Split (Random)**	0.8284 (0.7986 - 0.8553)	0.8151 (0.7826 - 0.8436)	0.8112 (0.7822 - 0.8398)	0.8130 (0.7810 - 0.8416)	0.8935 (0.8707 - 0.9153)	0.8235 (0.7708 - 0.8737)
**Patient-level Split (Strict Isolation)**	0.8300 (0.8014 - 0.8557)	0.8184 (0.7862 - 0.8476)	0.8189 (0.7863 - 0.8484)	0.8187 (0.7874 - 0.8481)	0.8875 (0.8618 - 0.9131)	0.8268 (0.7785 - 0.8704)

Note: Data are presented as values with 95% Confidence Intervals obtained via 1,000 bootstrap iterations. This ablation study was conducted on a well-annotated subset of the BabyEar4K dataset (comprising 2,136 normal ears and 1,385 abnormal ears including 658 lop, 264 Stahl's, and 463 helical deformities) where explicit bilateral identifiers (left/right ears of the same neonate) were available. Both models were trained for 15 epochs under an 8:2 train-test split. The patient-level split ensures that paired ears from the same neonate are strictly isolated within either the training or testing set, completely preventing patient-specific data leakage.

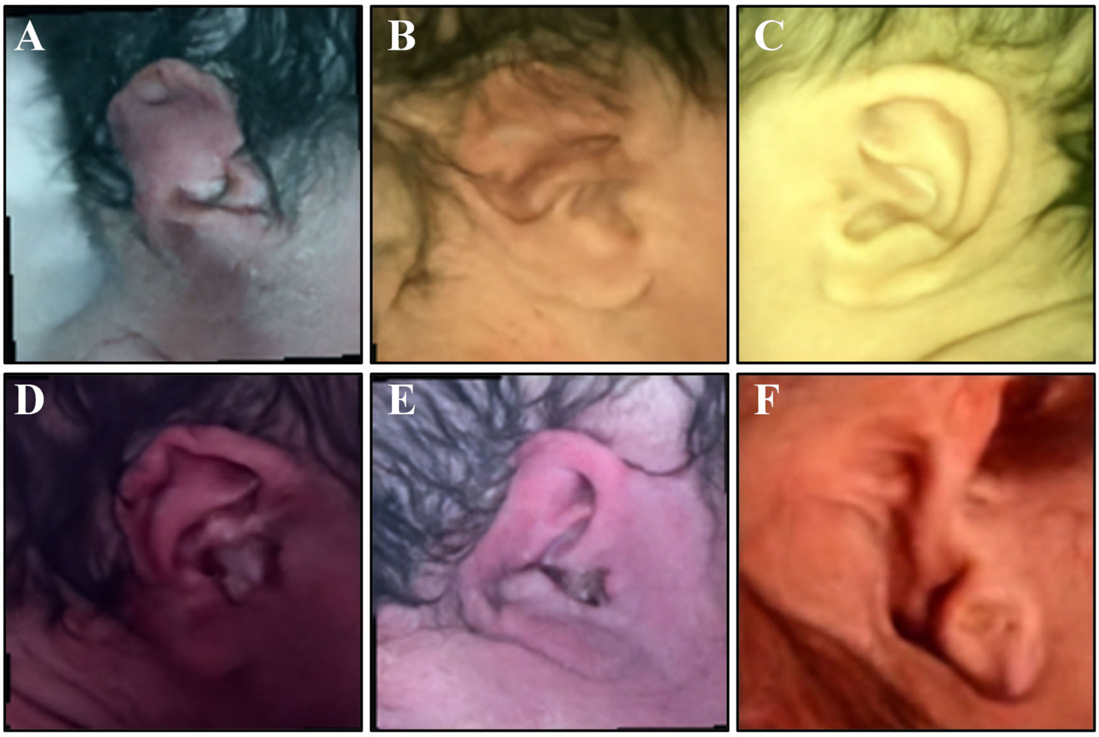

Figure S1. Representative failure cases of synthetic images generated by the Denoising Diffusion Probabilistic Model. These images illustrate typical generation artifacts that failed the expert review criteria and were subsequently excluded from the final synthetic stress test set. Common failure modes include severe anatomical distortions, surreal blurring of structural boundaries, unnatural skin textures, and unidentifiable morphologic hallmarks.

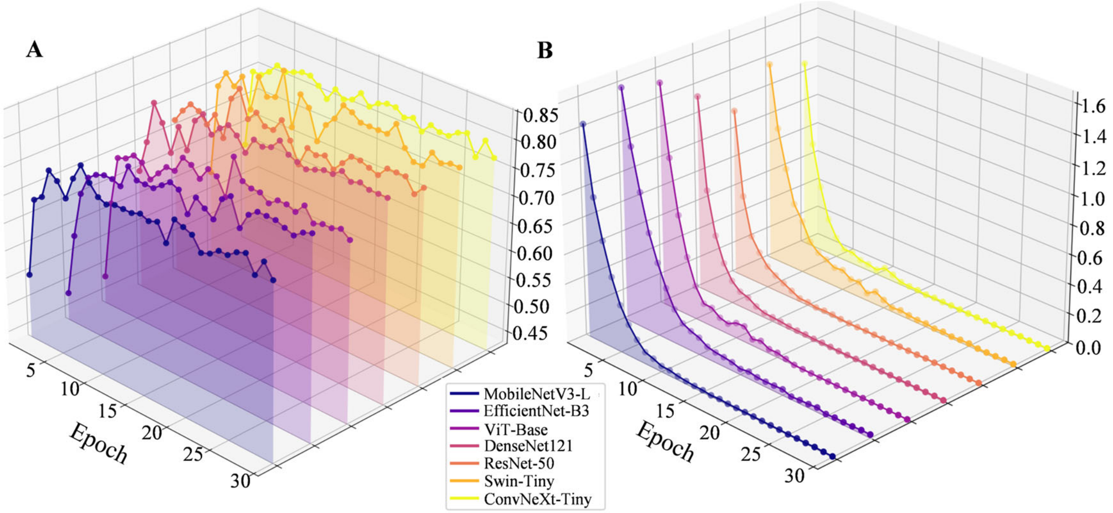

Figure S2. Training dynamics of different deep learning backbones in the intelligent diagnosis task of neonatal ear deformities. (A) 3D line plot of validation accuracy varying with training epochs for the six-class task. This panel presents the evolution trend of validation accuracy for the seven compared models over 30 training epochs, demonstrating the learning and generalization processes of each model. (B) 3D line plot of training loss varying with training epochs for the six-class task. It records the descent process of the training loss for the models over 30 epochs, where the smoothness and descent speed intuitively reflect the optimization efficiency and feature-learning stability of different backbones in this diagnosis task.

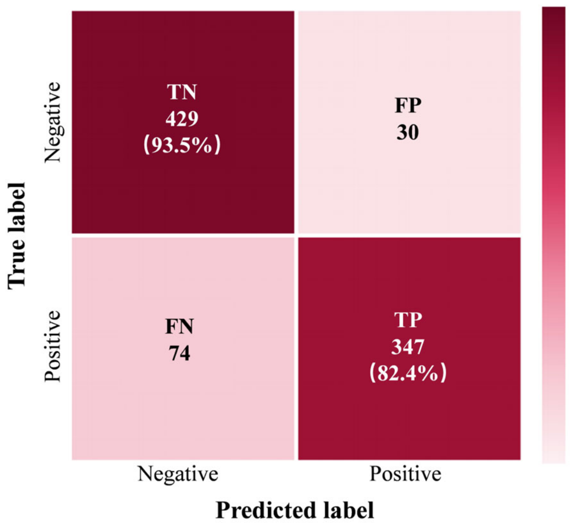

Figure S3. Normalized confusion matrix of the binary classification task (normal vs. deformed) on the test set. Rows and columns denote true and predicted classes, respectively. Diagonal cells indicate the counts and percentages of correct classifications (TN: true negative; TP: true positive), while off-diagonal cells show misclassifications (FP: false positive; FN: false negative). The color scale represents normalized proportions.

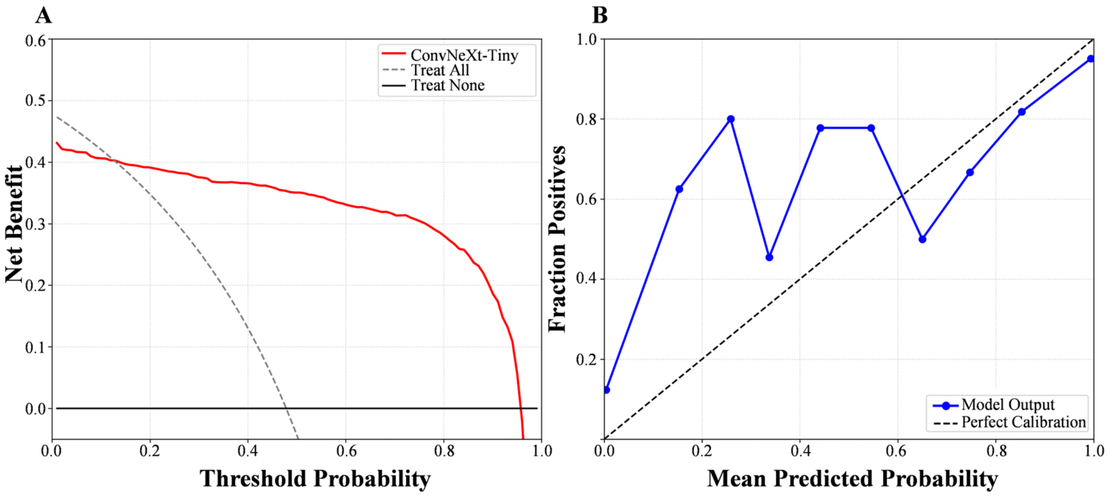

Figure S4. Clinical utility and probability calibration of the binary screening model. (A) Decision Curve Analysis (DCA). This curve demonstrates the net clinical benefit, showing that the ConvNeXt-Tiny model maintains consistently higher benefits across varying threshold probabilities compared to the default "Treat All" or "Treat None" strategies. (B) Calibration curve. This plot illustrates the reliability of the model's risk predictions by comparing the mean predicted probabilities against the actual fraction of positive cases. The dashed and solid black lines represent theoretical baselines for extreme treatment strategies and perfect calibration, respectively.

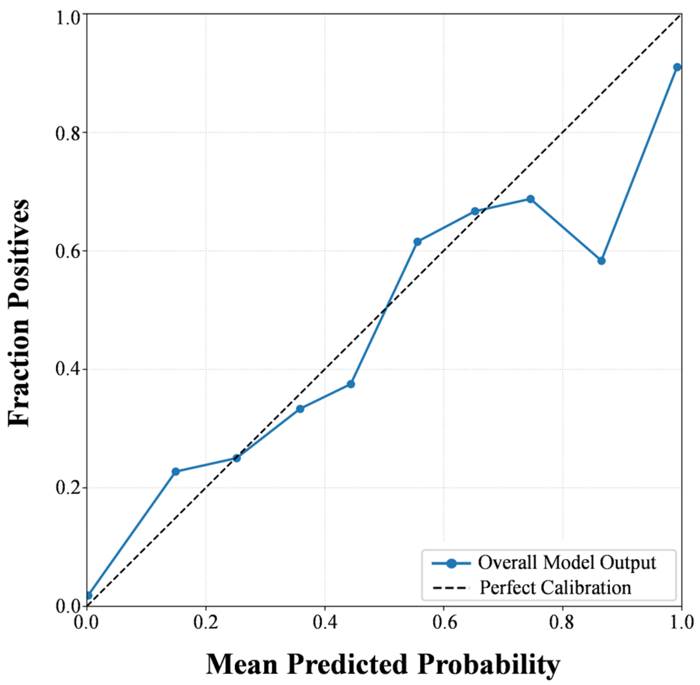

Figure S5. Overall calibration curve for the six-class deformity subtype identification model. The curve was computed using an overall flattened approach across all six subtypes on the internal test set. The solid blue line represents the empirical model output, and the dotted black line represents perfect theoretical calibration.

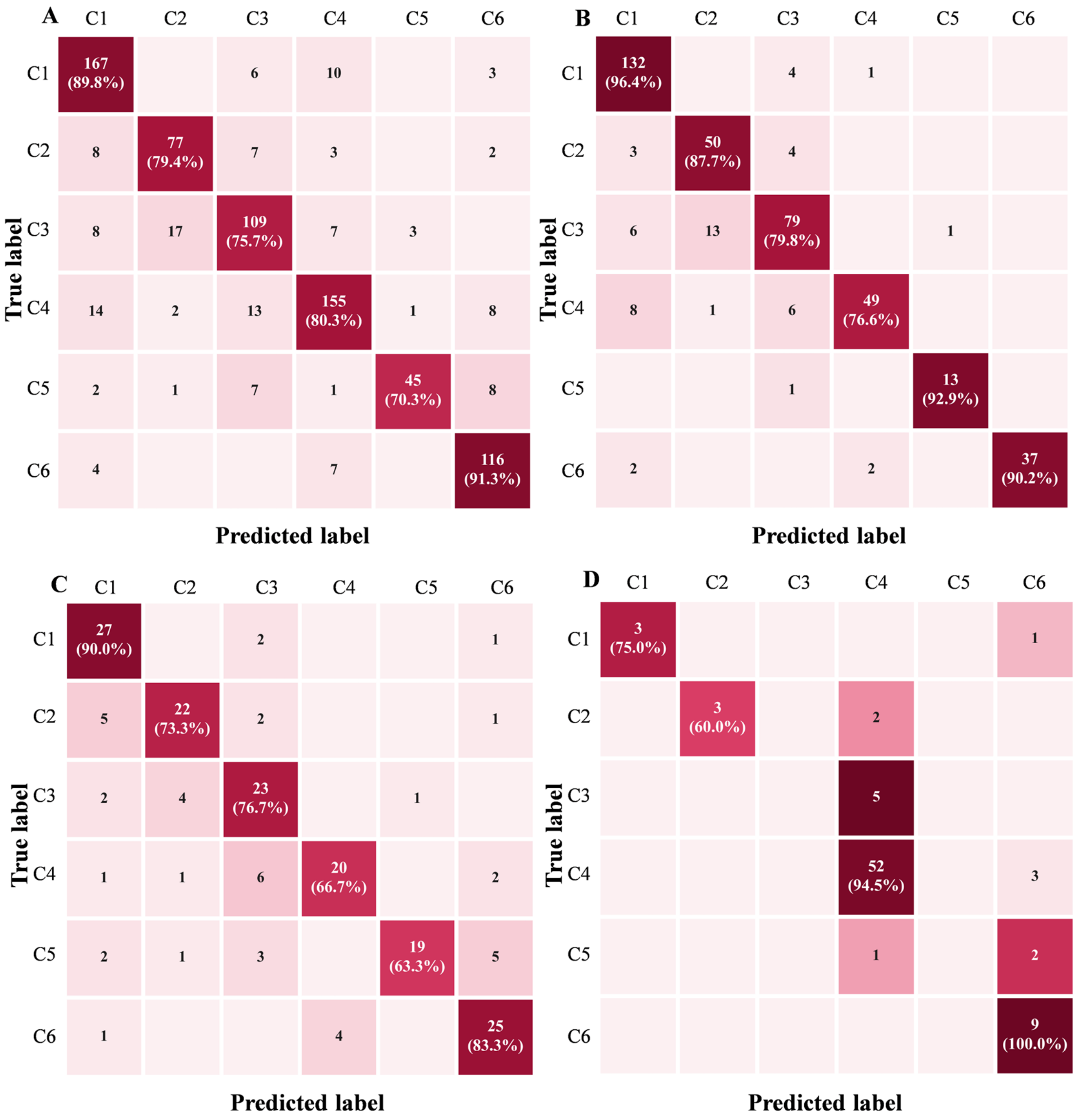

Figure S6. Confusion matrices for the aggregated and individual test sets. (A) Aggregated confusion matrix. This matrix compiles all 811 instances across the four evaluation sets to serve as an overall reference for systematic error pathways. (B) Internal test set confusion matrix. This matrix reflects the optimized baseline diagnostic performance of our model (n=412). (C) Synthetic stress test set confusion matrix. This panel evaluates model robustness on balanced rare subtypes generated via the DDPM (n=180). (D) Prospective clinical test set confusion matrix. This matrix assesses the generalization capability of our system in real world clinical scenarios (n=81). Rows and columns denote true and predicted classes, respectively (C1 to C6: Lop Ear, Stahl's Ear, Helical Deformity, Cup/Constricted Ear, Cryptotia, and Microtia). Diagonal cells indicate the counts and percentages of correct classifications, with the color scale representing normalized proportions.

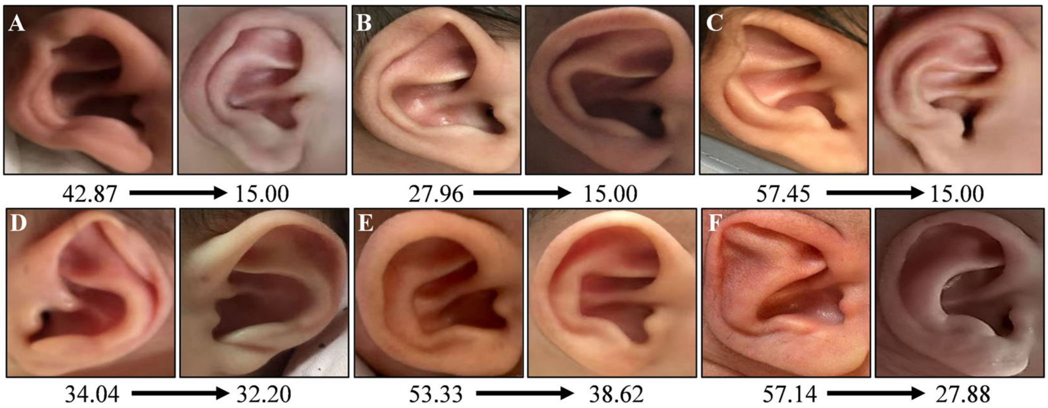

Figure S7. Clinical tracking of neonatal ear deformities pre- and post-treatment. (A-C) Representative successful cases achieving morphological normalization post-treatment, with scores decreasing below the threshold. (D-F) Atypical cases or those exhibiting partial improvement, where residual deformities persist despite score reductions. Values below the images indicate the automated deformity scores generated by the model.

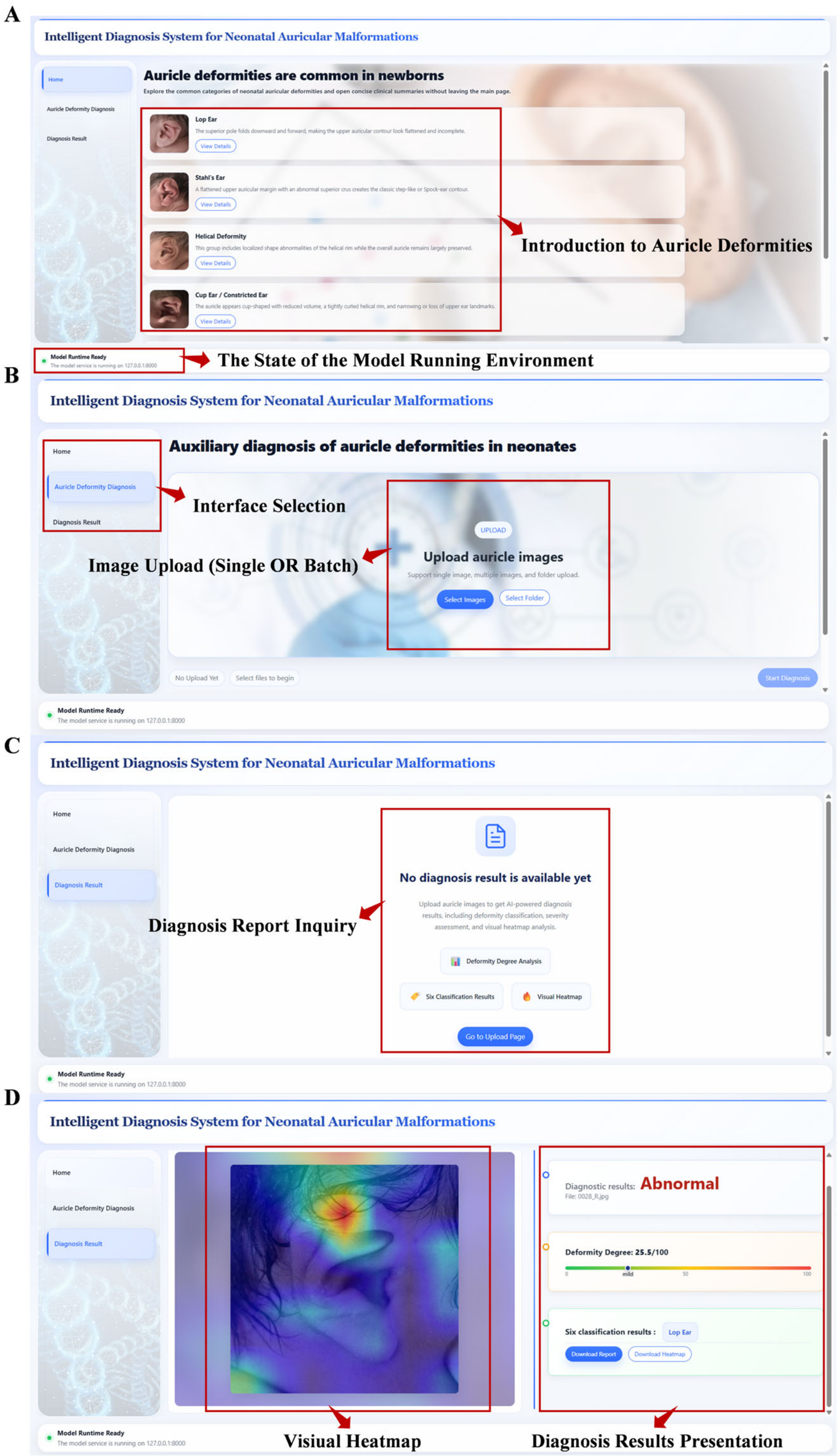

Figure S8. The web-based intelligent auxiliary diagnosis system interface. (A) The main homepage. This page provides access to educational resources on common auricular deformities. (B) The diagnostic interface. This interface supports single or batch upload of ear images for one-click diagnostic evaluation. (C) The diagnostic history page. This page allows users to review historical diagnostic results and corresponding Grad-CAM heatmaps. (D) A representative diagnostic results display page. This page presents the predicted binary screening outcome, severity score (displayed as "Deformity Degree"), six-class subtype classification and the associated heatmap visualization for a single deformed ear.

Supplementary Text 1: Anatomical Basis for Systematic Misclassifications

Based on our study findings, we observed that certain deformity subtypes are prone to systematic misclassification. These overlaps predominantly occur between subtypes sharing ambiguous anatomical boundaries and continuous morphological spectra. The anatomical rationales for the three most frequently confounded pathways are detailed below:

1. Stahl's Ear vs. Helical Deformity

Stahl's ear is defined by an abnormal third crus that often severely distorts the helical contour. The algorithm tends to disproportionately focus on this secondary helical irregularity rather than the primary cartilaginous etiology (the third crus), leading to a misclassification of helical deformity.

2. Lop Ear vs. Cup/Constricted Ear

These two deformities exist on a continuous morphological spectrum affecting the superior pole of the auricle. Their shared visual hallmark （an overhanging superior helical rim） makes establishing a discrete decision threshold between a severe lop ear and a mild cup ear inherently challenging.

3. Cup/Constricted Ear vs. Helical Deformity

Mild cup ears often exhibit tight, rolled-in helical margins (lidding) without pronounced global size reduction. The model frequently overweighs these localized rim abnormalities, conflating the specific helical tightness of a cup ear with primary isolated helical deformities.

Conclusion

In summary, these systematic errors reflect intrinsic anatomical ambiguities rather than random computational noise, underscoring the inherent clinical subjectivity in discretely subtyping morphologically continuous anomalies.
